# Lifestyle weight-loss intervention may attenuate methylation aging: the CENTRAL MRI randomized controlled trial

**DOI:** 10.1186/s13148-021-01038-0

**Published:** 2021-03-04

**Authors:** Anat Yaskolka Meir, Maria Keller, Stephan H. Bernhart, Ehud Rinott, Gal Tsaban, Hila Zelicha, Alon Kaplan, Dan Schwarzfuchs, Ilan Shelef, Yftach Gepner, Jun Li, Yifei Lin, Matthias Blüher, Uta Ceglarek, Michael Stumvoll, Peter F. Stadler, Meir J. Stampfer, Peter Kovacs, Liming Liang, Iris Shai

**Affiliations:** 1 Department of Public Health, Faculty of Health Sciences, Ben-Gurion University of the Negev, 84105 Beer-Sheva, Israel; 2 Helmholtz Institute for Metabolic, Obesity and Vascular Research (HI-MAG) of the Helmholtz Center Munich at the University of Leipzig and University Hospital Leipzig, Leipzig, 04103 Germany; 3 Medical Department III – Endocrinology, Nephrology, Rheumatology, University of Leipzig Medical Center, 04103 Leipzig, Germany; 4 Interdisciplinary Center for Bioinformatics, University of Leipzig, 04107 Leipzig, Germany; 5 Bioinformatics Group, Department of Computer Science, University of Leipzig, 04107 Leipzig, Germany; 6 Transcriptome Bioinformatics, LIFE Research Center for Civilization Diseases, University of Leipzig, 04107 Leipzig, Germany; 7 Soroka University Medical Center, Beer-Sheva, 84101 Israel; 8 Department of Epidemiology and Preventive Medicine, School of Public Health, Sackler Faculty of Medicine and Sylvan Adams Sports Institute, Tel Aviv University, Tel Aviv, 6997801 Israel; 9 Department of Nutrition, Harvard T.H. Chan School of Public Health, Boston, 02115 MA USA; 10 Department of Epidemiology, Harvard T.H. Chan School of Public Health, Boston, MA 02115 USA; 11 Institute for Laboratory Medicine, University of Leipzig Medical Center, Leipzig, 04103 Germany; 12 Deutsches Zentrum Für Diabetesforschung, Neuherberg, 85764 Germany; 13 Competence Center for Scalable Data Services and Solutions Dresden/Leipzig, German Centre for Integrative Biodiversity Research (iDiv), and Leipzig Research Center for Civilization Diseases, University of Leipzig, 04109 Leipzig, Germany; 14 Max Planck Institute for Mathematics in the Sciences, 04103 Leipzig, Germany; 15 Fraunhofer Institute for Cell Therapy and Immunology, 04103 Leipzig, Germany; 16 Department of Theoretical Chemistry, University of Vienna, 1090 Vienna, Austria; 17 Center for RNA in Technology and Health, University of Copenhagen, 1871 Frederiksberg, Denmark; 18 Santa Fe Institute, Santa Fe, NM 87501 USA; 19 Channing Division of Network Medicine, Department of Medicine, Brigham and Women’s Hospital and Harvard Medical School, Boston, 02115 MA USA; 20 Department of Biostatistics, Harvard T.H. Chan School of Public Health, Boston, 02115 MA USA

**Keywords:** Age prediction, Intrahepatic fat, DNA methylation, Aging, Weight loss

## Abstract

**Background:**

DNA methylation age (mAge), a methylation biomarker for the aging process, might serve as a more accurate predictor of morbidity and aging status than chronological age. We evaluated the role of multiple factors, including fat deposition, cardiometabolic risk factors and lifestyle weight-loss intervention, on the deviation of mAge from chronological age (mAge deviation) or 18-month change in mAge (∆mAge). In this sub-study of the CENTRAL magnetic resonance imaging weight-loss trial, we evaluated mAge by a validated 240-CpG-based prediction formula at baseline and after 18-month intervention of either low fat (LF) or mediterranean/low carbohydrate (MED/LC) diets.

**Results:**

Among 120 CENTRAL participants with abdominal obesity or dyslipidemia, mAge (mean ± SD: 60.3 ± 7.5 years) was higher than the chronological age (48.6 ± 9.3 years) but strongly correlated (*r* = 0.93; *p* = 3.1 × 10^–53^). Participants in the lowest tertile of mAge deviation from their chronological age had significantly lower waist-circumference, visceral adipose tissue, intrahepatic fat (IHF) content, fasting-glucose and HOMA-IR, as compared with participants in the highest sex-specific residual tertile (*p* < 0.05 for all). IHF% remained associated with greater mAge deviation after further adjustments (*β* = 0.23; *p* = 0.02). After 18-month weight-loss lifestyle intervention, mAge remained significantly correlated with chronological age (*r* = 0.94, *p* = 1.5 × 10^–55^). mAging occurred, with no difference between lifestyle intervention groups (∆ = 0.9 ± 1.9 years in MED/LC vs. ∆ = 1.3 ± 1.9 years in LF; *p* = 0.2); however, we observed a mAging attenuation in successful weight losers (> 5% weight loss) vs. weight-loss failures ( ∆ = 0.6 years vs. ∆ = 1.1 years; *p* = 0.04), and in participants who completed the trial with healthy liver fat content (< 5% IHF) vs. participants with fatty liver (∆ = 0.6 years vs. ∆ = 1.8 years; *p* = 0.003). Overall, 18 months of weight-loss lifestyle intervention attenuated the mAging of the men, mainly the older, by 7.1 months than the expected (*p* < 0.05).

**Conclusions:**

Lifestyle weight-loss intervention may attenuate mAging. Deviation of mAge from chronological age might be related to body fat distribution and glycemic control and could indicate biological age, health status and the risk for premature cardiometabolic diseases.

*Trial registration*: ClinicalTrials.gov NCT01530724. Registered 10 February 2012, https://clinicaltrials.gov/ct2/show/study/NCT01530724.

**Supplementary Information:**

The online version contains supplementary material available at 10.1186/s13148-021-01038-0.

## Background

Epigenetic modifications include changes on the genome that may alter gene expression without changing the DNA sequence [1]. Epigenetic alterations might be induced by several factors such as genetics [2], environmental [3] and lifestyle factors [4, 5]. Importantly, they were suggested as mediators to aging and lifespan-related conditions [6]. A major mechanism underlying epigenetic regulation is the DNA methylation at cytosine followed by guanines (CpG sites) [7], where DNA methyltransferases are responsible for the methylation in the CpG region [8]. Prediction of age by DNA methylation of specific CpGs was first described by Horvath [9], followed by others [10, 11], as aging was associated with DNA methylation [12].

Prediction of age by methylation level at specific sites has been performed among several populations (which differed mainly by age and/or race), by different prediction formulas, described as “epigenetic clock,” “age acceleration” (expressing the difference between age predicted by DNA methylation and chronological age) or “methylation age” (mAge). These formulas successfully predicted age by an epigenetic features [9–11]. mAge, as evaluated by specific CpGs formulas [9], is likely to increase at a slower pace than actual age across the life course, especially in older populations [13]. Methylation aging, the deviation between mAge and chronological age, or age acceleration, are suggested to be strong predictors of all-cause mortality [12, 14] and fatal and nonfatal cardiovascular disease [15–18]. In another epigenetic clock study, using Horvath’s 353 CpG-based formula, epigenetic aging rates were significantly associated with sex, race/ethnicity and coronary heart disease risk factors [19]. mAge was also higher with greater air pollution [11] and body mass index (BMI)[20].

Caloric restriction, with no specific dietary pattern, has also been demonstrated to halt aging by attenuating age-associated epigenetic alterations [21]. In a 16-week intervention trial, vitamin D3 supplementation among 70 overweight and obese individuals led to slower epigenetic aging [22]. It has been suggested the Mediterranean (MED) diet might increase lifespan and improve aging [23] due to its unique combination of fatty acids, antioxidants, vitamins and phytochemicals. However, findings regarding the association of lifestyle factors, such as smoking, physical activity and diet, with mAge are inconsistent or marginal [20, 24] and mostly shown in longitude and cross-sectional studies.

The relationship of mAge with ectopic fat accumulation is unknown. Body fat deposits are strong indicators of metabolic state and cardiometabolic risk [25]. Visceral adipose tissue (VAT) is a prominent predictor of type 2 diabetes mellitus, cardiovascular disease and reflects the extent of other ectopic fats as in the liver, heart and pancreas [26, 27]. Increased liver fat is associated with metabolic syndrome [28–32] and insulin resistance [33]. We have reported the 18-month CENTRAL MRI trial results, in which we assessed the differential mobilization of VAT, intrahepatic fat (IHF) and other specific fat depots by different lifestyle interventions, linking the changes to specific clinical biomarkers [34]. In this sub-study of the CENTRAL trial, we aimed to explore the relation of mAge (calculated by a 240-CpG-based prediction formula) and its changes with IHF and abdominal fat deposits, anthropometric parameters and blood biomarkers reflecting cardiometabolic risk (e.g., glycemic and lipid markers) among abdominally obese participants undergoing a weight-loss intervention.

## Results

### Baseline characteristics

At baseline, the mean chronological age was 48.3 ± 9.5 years for men (*n* = 110) and 51.5 ± 6.9 for women (*n* = 10). Mean mAge was 60.1 ± 7.6 years for men and 62.4 ± 6.3 years for women. The participants had a mean waist circumference (WC) of 107.2 ± 7.1 cm for men and 101.7 ± 15.0 cm for women. Abdominal fat proportion was distributed between genders as follows: VAT: 35.4 ± 10.1% vs. 22.2 ± 4.8% (*p* = 9.9 × 10^–5^), deep subcutaneous adipose tissue (SAT): 40.1 ± 6.1% vs. 38.9 ± 6.2% (*p* = 0.57), superficial SAT: 24.4 ± 5.9% vs. 38.9 ± 6.2% (*p* = 2.0 × 10^–6^; all men vs. women, respectively). 58.8% of the participants were defined with fatty liver (> 5% IHF), with no difference in IHF% between sex groups were observed (10.6 ± 10.1 and 11.6 ± 16.0 men vs. women; *p*  = 0.52). No significant baseline differences were observed between intervention groups in mAge (*p* = 0.77 for low fat (LF) vs. MED/low carbohydrates (MED/LC)) or age (*p* = 0.69).

**Fig. 1:**
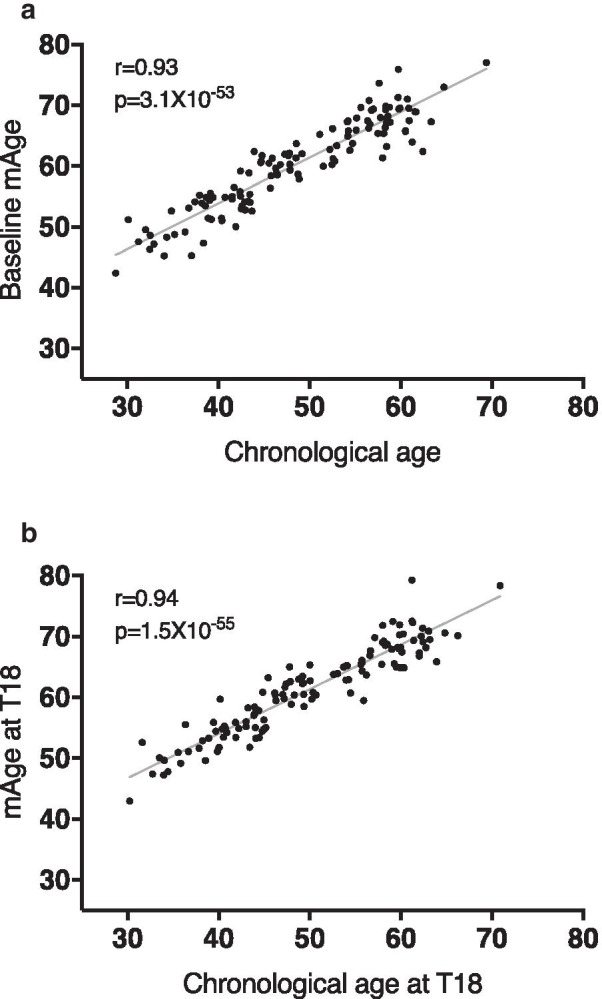
Correlation between baseline age and mAge. **a** Baseline correlation between age and mAge. **b** Correlation between age and mAge after 18 m of lifestyle intervention

At baseline, chronological age was strongly correlated with mAge (*r* = 0.93, *p* = 3.1 × 10^–53^, Fig. 1a). Chronological age was negatively correlated with weight (*r* = − 0.21, *p* = 0.02) and with deep SAT proportion (*r* = − 0.29, *p* = 0.002), superficial SAT area and proportion (*r* = − 0.26, *p* = 0.004; *r* = − 0.37, *p* = 3.5 × 10^–5^) and positively correlated with VAT area and proportion (*r* = 0.38, *p* = 1.9 × 10^–5^; *r* = 0.42,* p* = 2.0 × 10^–6^). Similarly, mAge was positively correlated with VAT proportion and area (*r* = 0.41; *p*  = 4.0 × 10^–6^; *r* = 0.38, *p* = 1.9 × 10^–5^) and negatively with deep SAT proportion (*r* = − 0.27, *p* = 0.003), superficial SAT area and proportion (*r* = − 0.23, *p* = 0.01; *r* = − 0.38, *p* = 2.3 × 10^–5^).

### Baseline associations of the mAge deviation with adiposity and blood biomarkers

Examining sex-specific tertiles of the mAge deviation, measured by standardized residuals from the predicted values (calculated using the 240-CpGs formula; Table 1), we observed that participants in the lowest tertile of mAge deviation from their chronological age had significantly lower WC (105.2 ± 6.8 cm vs. 109.4 ± 8.3 cm), VAT area (166.3 ± 50.4cm^2^ vs. 196.4 ± 69.0 cm^2^) and IHF% content (8.3 ± 8.0% vs. 14.5 ± 12.8%), as compared with participants in the highest sex-specific residual tertile. Out of all the examined blood array of biomarkers, only glycemic biomarkers (e.g., fasting glucose) were significantly different between mAge deviation tertiles (*p* < 0.05 for all). (Table 1). A sensitivity analysis among men only (92% of participants) is presented in Additional file 1: Table S1.

**Table 1:** Baseline characteristics of the CENTRAL participants across sex-specific tertiles of mAge deviation

	Entire *n* = 120	Low tertile *n* = 40	Intermediate tertile *n* = 41	High tertile *n* = 39	*p* between tertiles^a^	*p* between extreme tertiles^b^
Age, years	48.6 ± 9.3	45.1 ± 10.2	50.7 ± 9.0	47.3 ± 8.4	0.19	0.92
mAge, years	60.3 ± 7.5	56.5 ± 7.5	62.2 ± 6.9	62.3 ± 6.8	**2.6 × 10**^**–4**^	**0.001**
Weight, kg	90.3 ± 11.4	88.3 ± 9.5	89.3 ± 12.9	92.9 ± 11.5	0.17	***0.06***
BMI, kg/m^2^	30.2 ± 3.3	29.8 ± 2.6	29.8 ± 3.4	30.9 ± 3.9	0.44	0.26
WC, cm	106.7 ± 8.1	105.2 ± 6.8	105.6 ± 8.6	109.4 ± 8.3	**0.03**	**0.008**
VAT, cm^2^	176.2 ± 61.2	166.3 ± 50.4	166.6 ± 59.8	196.4 ± 69.0	*0.07*	**0.04**
VAT proportion, %	34.3 ± 10.5	33.8 ± 10.7	33.5 ± 9.7	35.8 ± 11.2	0.59	0.44
DSAT, cm^2^	210.9 ± 70.0	204.3 ± 64.7	210.0 ± 78.4	218.6 ± 66.9	0.70	0.42
DSAT proportion, %	40.0 ± 6.1	39.8 ± 6.1	40.1 ± 5.8	39.4 ± 6.3	0.57	0.79
SSAT, cm^2^	134.9 ± 56.5	136.3 ± 55.6	130.0 ± 51.5	138.5 ± 63.2	0.79	0.87
SSAT proportion, %	25.6 ± 7.1	26.4 ± 7.3	25.7 ± 7.3	24.8 ± 6.9	0.63	0.33
IHF^c^, %	10.71 ± 10.6	8.3 ± 8.0	9.3 ± 9.8	14.5 ± 12.8	**0.03**	**0.02**
Fasting glucose, mg/dL	106.5 ± 16.2	100.8 ± 10.3	110.1 ± 18.9	108.4 ± 16.9	**0.02**	**0.01**
HOMA IR^d^	4.8 ± 3.5	3.9 ± 2.1	4.7 ± 3.5	5.9 ± 4.3	*0.08*	**0.03**
HbA1c, %	5.6 ± 0.5	5.5 ± 0.4	5.7 ± 0.5	5.6 ± 0.5	0.29	0.47

Data are mean ± SD. ^a^ANOVA or Kruskal Wallis. ^b^T-test or Mann–Whitney. ^c^*n* = 119. ^d^*n* = 114. Lowest tertile: men: ≤ − 0.44; women ≤ − 0.77; intermediate tertile: men: − 0.43 to 0.46, women: − 0.76 to 0.55; Highest tertile: men: 0.47+, women: 0.56+. Significant associations (*p* < 0.05) are represented in bold. Non-significant associations with *p* < 0.1 are represented in italics

*BMI* body mass index, *DSAT* deep subcutaneous adipose tissue, *HbA1c* hemoglobin A1c, *HOMA IR* homeostatic model assessment of insulin resistance, *IHF* intrahepatic fat, *SSAT* superficial subcutaneous adipose tissue, *VAT* visceral adipose tissue, *WC* waist circumference

In multiple linear regression models (Table 2), greater mAge deviation remained significantly associated with increased IHF% (sex, weight and age adjusted model: *β* = 0.23, *p* = 0.02). Fasting glucose remained marginally associated with mAge deviation.

**Table 2:** Multivariate models for the assessment of mAge deviation with adiposity and glycemic markers

	Model 1		Model 2		Model 3	
	*β*	*p* value	*β*	*p* value	*β*	*p* value
Weight, kg	0.05	0.62	–	–	–	–
BMI, kg/m^2^	0.09	0.35	*	*	*	*
WC, cm	0.14	0.13	*	*	*	*
VAT, cm2	0.15	0.12	0.14	0.14	0.18	0.11
DSAT, cm^2^	0.07	0.48	0.06	0.63	0.06	0.63
SSAT, cm^2^	0.04	0.97	− 0.05	0.73	− 0.05	0.74
IHF, %	**0.3**	**0.02**	**0.23**	**0.02**	**0.23**	**0.02**
Fasting glucose, mg/dL	*0.16*	***0.08***	*0.16*	***0.08***	*0.18*	***0.07***
HOMA IR	0.15	0.12	0.14	0.16	0.15	0.15
HbA1c, %	0.06	0.49	0.07	0.48	0.07	0.48

*Cannot be tested in a multivariate model due to collinearity of weight with WC/BMI. *BMI* body mass index, *DSAT* deep subcutaneous adipose tissue, *HbA1c* hemoglobin A1c, *HOMA IR* homeostatic model assessment of insulin resistance, *IHF* intrahepatic fat, *SSAT* superficial subcutaneous adipose tissue, *VAT* visceral adipose tissue, *WC* waist circumference. Significant associations (*p* < 0.05) are represented in bold. Non-significant associations with *p* < 0.1 are represented in italics

Model 1: Standardized residuals—adjusted for sex

Model 2: Standardized residuals—adjusted for sex and weight

Model 3: Standardized residuals—adjusted for sex, weight and age

Since IHF% and fasting glucose showed significant/marginal association in all three models, we used further adjustment to lifestyle factors (smoking or alcohol intake). These did not attenuate the associations with IHF. Furthermore, adding daily alcohol intake resulted in a significant association with mAge deviation in all three models for fasting glucose. Of note, no differences in mAge or mAge deviation between smokers and non-smokers were observed (*p* = 0.51 and *p* = 0.48), and no correlation between mAge or mAge deviation with alcohol intake were found (*r* = 0.12, *p* = 0.2; *r* = − 0.11, *p* = 0.24).

Next, we used the three models presented in Table 2 to examine the association between mAge deviation and IHF% and added medical conditions as the presence of type 2 diabetes or metabolic syndrome. These models remained significant and fully reported in Additional file 2: Table S2.

### Evaluation of baseline results using 353-CpG-based mAge formula

Next, we re-examined our data in a 353-CpG-based mAge prediction formula; the first mAge formula described, well based and commonly used named the “Horvath clock” [9]. A significant correlation was observed between chronological age and the 353-CpG-based mAge (*r* = 0.90, *p* = 3.5 × 10^–44^). 353-CpG-based mAge was also correlated with superficial SAT area (*r* = − 0.23, *p* = 0.01) and proportion (*r* = − 0.45, *p* = 2.4 × 10^–7^) as well as with VAT area (*r* = 0.37, *p* = 3.6 × 10^–5^) and proportion (*r* = 0.42, *p* = 1.0 × 10^–6^) and deep SAT proportion (*r* = − 0.24, *p* = 0.007). A summarize of the baseline correlations of mAge with adiposity and fat deposits according to two mAge prediction formulas is presented in Additional file 3: Table S3. Also, a marginal difference was observed for IHF% tertiles (*p* = 0.089), with IHF levels among participants in the lower sex-specific mAge deviation tertile (mean mAge deviation ≤ − 0.4 for men and ≤ − 0.53 for women) significantly lower (*n* = 38; 8.01 ± 8.8%), as compared with IHF% in the highest sex-specific tertile (*n* = 41; men: ≥ 0.41; women: ≥ − 0.21; mean IHF% of 12.1 ± 10.3%; *p*  = 0.03).

### The effect of 18-month lifestyle intervention on mAge change (∆mAge, based on 240-CpGs formula)

After 18 months of lifestyle intervention, the entire cohort had a mean weight loss of 4%, relative VAT change of − 26.2 ± 17%, and relative IHF% change of − 18.3 ± 84.1% (*p* < 0.05 vs. baseline for all). The mean mAge increased significantly by 1.1 ± 1.9 years and remained strongly correlated with chronological age (*r* = 0.94, *p* = 1.5 × 10^–55^) (Fig. 1b). We could not detect any significant associations between changes in adiposity parameters or blood biomarkers with a change in mAge.

The change of mAge did not differ significantly across the intervention groups: the mean mAge in the MED/LC group increased by 0.9 ± 1.9 years and 1.3 ± 1.9 years in the LF group, with no significant differences between groups in mAge change (*p* = 0.2) or weight loss (*p* = 0.47). Of note, a similar finding was observed for the four intervention groups in terms of within-group increase from baseline (LF: + 1.2 ± 1.9, *p* = 0.002; MED/LC: + 1.0 ± 1.9, *p* = 0.006; LF + PA: + 1.4 ± 2.0, *p* = 0.001; MED/LC + PA: + 0.7 ± 1.9, *p* = 0.045) and between groups (*p* = 0.57). Further subgroup analysis by chronological age is presented in Additional file 4: Supplementary results.

**Fig. 2:**
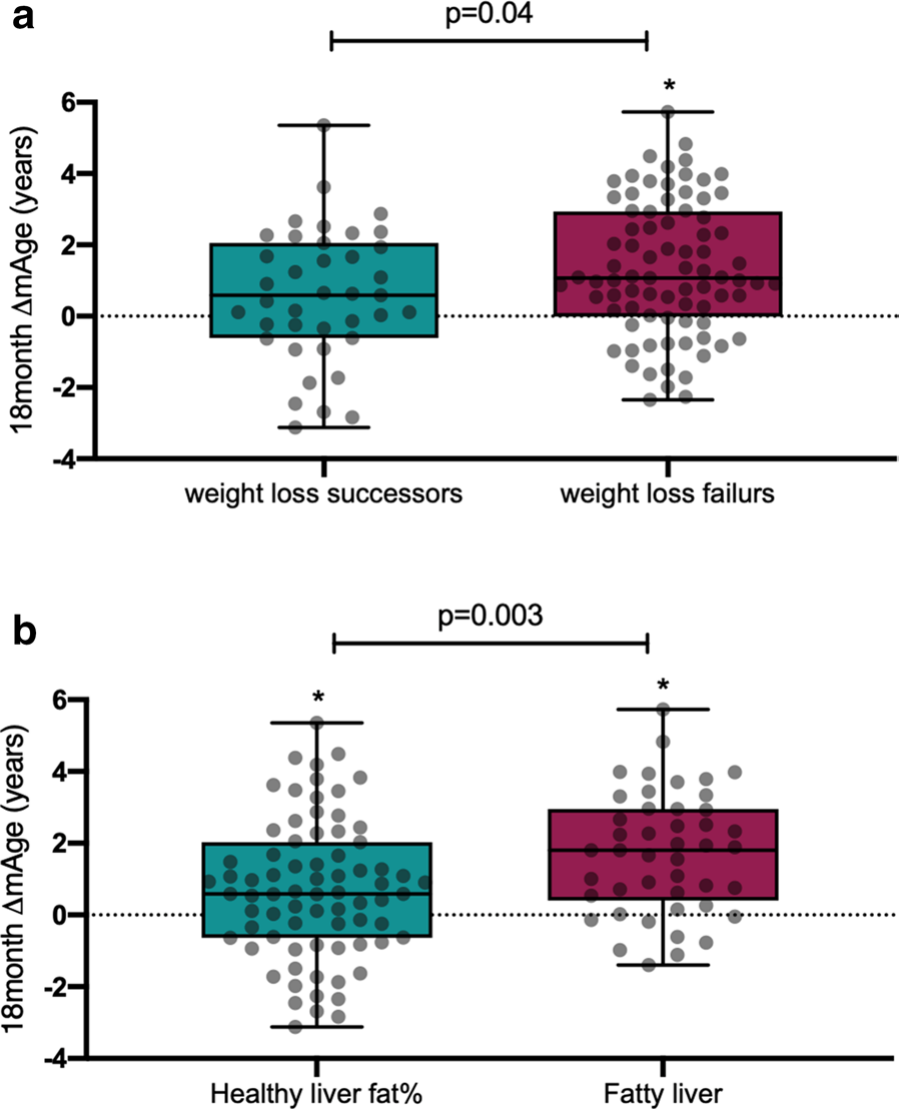
18-month change in mAge between subgroups. **a** Weight-loss successors (*n* = 39) vs. weight-loss failures (*n* = 81). **b** participants with healthy liver fat% at the end of the intervention (*n* = 75) vs. fatty liver (*n* = 45 for fatty liver). Boxplots whiskers represent min to max. * denotes within-group difference (T18 vs. T0). Dots represent individual values

Among successful weight-loss responders (those who lost more than 5% of initial body weight, 32.5% of our cohort, mean weight loss of − 9.6%), the median increase of mAge was 0.6 years (25^th^,75^th^ percentiles: − 0.61, 2.1), was significantly lower than the median change of 1.1 years (− 0.02, 2.9) among the weight-loss failures (those who lost less than 5% or gained weight; − 1.2% weight loss), *p* = 0.04 between groups (Fig. 2a). Further adjustment for sex, baseline mAge and 18-month weight loss did not attenuate the significant difference (*p* = 0.04). A similar multivariate model, excluding baseline mAge, yielded similar results (*p* = 0.03).

**Fig. 3:**
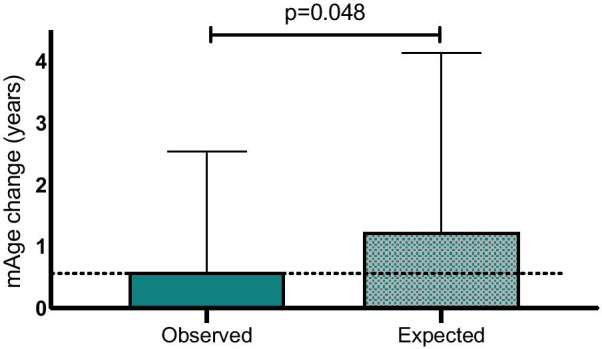
The observed mAge change following the intervention vs. the expected mAge change among men above median age. The observed mAge change is the actual difference observed between timepoints for this subgroup. The expected mAge change was calculated using the linear regression prediction formula generated from baseline correlation between age and mAge. Data presented as means and SDs

Participants with IHF% under 5% at the end of the intervention (“healthy liver status”; mean IHF of 3.3%, Fig. 2b) had significantly lower 18-month mAge increase of 0.6 years (− 0.64, 2.0), as compared with a median change of 1.8 years (0.4, 3.0) among participants with fatty liver (IHF at the end of the intervention of > 5%; mean IHF of 7.3%), *p* = 0.003 between groups. This significant difference remained after further adjustment for age, sex, baseline mAge and 18-month IHF% loss (*p* = 1.6 × 10^–4^). The association remained significance after excluding baseline mAge from the multivariate model (*p* = 0.002). Observed mAge change (∆mAge) in relation to the predicted mAge change according to baseline linear prediction equation is presented in Additional file 4: Supplementary results and Additional file 5: Figure S1. Among older men (above the median age of 48 years), the observed mAge was significantly lower than the assumed expected (*p* = 0.048), where mAge increased by 7.1 ± 23.4 months from baseline, while the expected mAge change was assumed as 14.8 ± 35.8 months (Fig. 3).

## Discussion

In this 18-month trial among 120 participants with abdominal obesity or dyslipidemia, we found that lifestyle weight-loss intervention may attenuate mAging. Deviation of mAge from chronological age might be related to body fat distribution and glycemic control and could indicate biological age, health status and the risk for premature cardiometabolic diseases.

This analysis had several limitations. We used a prediction formula that was previously tested and validated only in normal-weight populations [11], as opposed to the participants of our study population who are abdominally obese. We reported sex-based differences despite having a potentially underpowered group of female sex due to low numbers. This low number of women participating in our trial reflects the low number of women in the workplace where we recruited our participants. Also, our array allowed the detection of only 217 of the 240 CpGs in the original prediction formula. Nonetheless, we observed a robust correlation between age and mAge, similar to one previously published across multiple populations of different ethnicities [11], with additional evaluation of the baseline results based on Horvath’s mAge prediction formula.

Aging is associated with changes in body composition (fat, muscle mass and bone density) [35], cognitive decline [36], decreased renal function [37], cancer [33] and more. In the past years, some biological age predictors (e.g., telomere length, metabolomics, specific blood biomarkers) were examined to find age-associated outcomes [39]. For this analysis, we used the 240 CpG-based formula that was previously trained and validated among 989 blood samples of the Chinese population and 160 Caucasians [11], both with weight within the normal range, with an excellent correlation coefficient (above 0.94). Among our 120 participants with abdominal obesity, we found a similar correlation between age and mAge both at baseline and at the end of the intervention. These results confirm our mAge prediction formula's ability to predict age in various populations and phenotypes accurately.

In our study, mAge was found to be associated with some abdominal fat depots, according to two mAge prediction calculations. For the mAge deviation, besides an association with WC, VAT and biomarkers of glycemia, which slightly attenuated after further adjustments, we observed that beyond sex, age, weight and lifestyle factors, participants with higher mAge than age had higher IHF%. There is much interest in exploring IHF levels since elevated triglycerides in the liver [“fatty liver” [40]] is a common reversible condition [mostly by weight reduction [41]]. Still, without proper treatment, it might progress to hepatocellular carcinoma [40, 42]. This finding could be due to other diseases' effects on these variables, e.g., hypertension or metabolic syndrome. However, due to this study's nature, we can only discuss associations and used adjustment in our models to reduce confounders, as presented in the results section. As most previous studies examined anthropometrically, blood markers and some diseases in relation to epigenetic age, we were able to add on current knowledge and examine some MRI assessed fat deposits, known to indicate health risks.

The CENTRAL study [25] was designed to examine fat mobilization following weight loss derived from lifestyle intervention strategies. As this one phase trial was conducted for 18 months, it is logical to assume that all participants aged both chronologically and biologically as time passed. Yet, some participants, and more specifically—weight-loss successors and healthy liver ones, demonstrated lower aging than others. Although we could not detect changes in mAge attenuation between diet groups, we observed significant differences between some groups, representing health status. We used thresholds for liver status and weight-loss success for this analysis according to previously published. A 5% weight loss is an apparently meaningful marker for health improvement, although greater weight loss might yield better health outcomes [43]. The liver status cut-off was set to 5% IHF, which is acceptable for fatty liver initial diagnosis with radiological imaging techniques [40]. With the understanding that aging, accompanied by age-related conditions and diseases, is inevitable, extensive research has been trying to find treatment or mechanism to halt age-related conditions, including dietary changes, as a potential strategy for slowing down aging. The CENTRAL study participants experienced a modest 18-month weight loss [25] following two types of calorie restriction strategies. Potentially, this weight loss (and other adverse advantages accompanied by this weight reduction, as a decrease in fatty liver status and improved cardiometabolic risk) improved aging among our participants. This evidence might promote a better understanding of the role of weight loss in improving life longevity. Yet, these results should be interpreted with caution since we cannot determine whether the weight loss was the main driver for the beneficial biological effect or the relief in cardiometabolic risk and/or the reductions in fatty liver prevalence.

## Conclusions

Methylation levels in specific CpGs can predict age in an overweight population. mAge deviation is associated with additional health traits, as fatty liver and impaired fasting glucose. mAge, measured by methylation level of specific CpGs, might serve as a biological marker for health. Weight loss and healthier liver might promote an increase in lifespan, reflected by this biological marker.

## Methods

### Study design and participants

The 18-month CENTRAL trial [clinicaltrial.gov identifier: NCT01530724] was conducted between October 2012 and April 2014 in a research center workplace in Dimona, Israel, and described previously in detail [25]. Recruitment began in May 2012, and by the start of the trial 278 sedentary individuals were eligible to participate. Inclusion criteria were: abdominal obesity [WC > 102 cm (40 inches) for men and > 88 cm (35 inches) for women], or serum triglycerides > 150 mg/dL and high-density-lipoprotein cholesterol (HDL-c) < 40 mg/dL for men and < 50 mg/dL for women. Exclusion criteria were: serum creatinine ≥ 2 mg/dL, impaired liver function (≥ threefold the upper level of Alanine transaminase and Aspartate transaminase), active cancer, pregnancy or lactation, highly physically active (> 3 h/week), or unable to take part in physical activity (PA), or participation in another trial. The study protocol was approved by the Medical Ethics Board and the Helsinki Committee of the Soroka University Medical Center. All participants provided written informed consent and received no financial compensation or gifts.

### Randomization and intervention

As previously described [25], participants were randomly assigned in two phases: first, two equally hypocaloric diets: a LF diet or a MED/LC diet. Second, after 6 months of dietary intervention, the two dietary groups were further randomized into diet only groups (LF, MED/LC) or groups with additional moderate physical activity intervention, mostly (80%) aerobic (LF + PA, MED/LC + PA).

### Magnetic resonance imaging and clinical measurements

A 45-min 3-T magnetic resonance imaging (MRI, Ingenia 3.0 T, Philips Healthcare, Best, the Netherlands) was used to scan all participants at baseline and after 18 months. Technical description of the scanning procedure, abdominal fat depots (VAT, DSAT, SSAT), IHF% acquisition and clinical measurements (anthropometric parameters, blood markers of glycemia and lipids, etc.) are available in Additional file 6: Supplemental methods.

### DNA sampling and extraction

Blood samples were taken after an overnight fast at baseline (T0) and 18 months (T18) after the individuals completed their interventions. Samples were stored at − 80 °C until DNA was extracted from peripheral blood samples following a standard protocol using proteinase K and 0.2% SDS at Hadassah Hebrew University Medical Center, Jerusalem. Samples were integrity-controlled using gel-electrophoresis, and the concentrations of double-stranded DNA were measured using Quant-iT PicoGreen dsDNA (Invitrogen, ThermoFisher Scientific, Germany) and Quantus (Promega, Germany) technologies.

### Sample selection and genome-wide DNA methylation

**Fig. 4:**
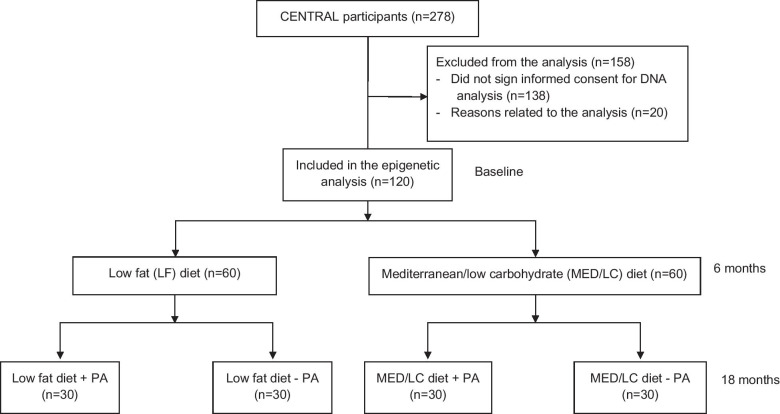
Flow diagram of the CENTRAL epigenetic cohort

This is a sub-study of the CENTRAL trial (Fig. 4), including 120 participants, according to the following criteria: both baseline and 18 months available blood samples and additional consent to genetic analysis. Sample selection was detailed elsewhere [44].

Five hundred ng of genomic DNA from each sample was bisulfite converted using EZ DNA Methylation Gold Kit (Zymo Research, Netherlands). Data were first assessed for quality at GenomeScan (Leiden, Netherlands) using the MethylAid package [45]. All quality control (QC) parameters were within predicted specifications, and more than 807.5 K sites (95%) were detected. Cell-type compositions were computed using minfi's estimate CellCounts function and the following cell types: CD8T cells, CD4T cells, NKcells, Bcells, Monocytes, Neutrophils. Datasets above or below 3 standard deviations from the mean relative amount (|z-score|> 3) in one cell type were excluded from further analysis. Following quality QC, amplification, and hybridization on Illumina MethylationEpic BeadChips (Illumina, Inc., San Diego, CA, U.S.A) the Illumina iScan array scanner was used to quantify genome-wide DNA methylation levels at ~ 850,000 CpG sites per sample on single-nucleotide resolution (GenomeScan, Leiden, Netherlands). Prior to all analysis steps aimed at identifying specific CpG sites (comparison independent), beta values were computed and quantile normalized using Minfi R package [46, 47]. DNA-Methylation analysis was performed at Leipzig University, Germany.

### Methylation age calculations

We used a prediction formula [11] based on methylation level at 240 specific CpGs sites, developed using whole blood samples and validated in multiple populations. To compute mAge, we used pre-quantile normalized beta values because the population mean and standard deviation for the CpG might be different from different populations. Out of the 240 CpGs in the formula, 217 were available after QC steps in our data: We multiplied each specific coefficient with our beta values for corresponding CpGs and summed up together to get the mAge before calibration (mAge BC). Finally, we computed mAge after calibration with the following: mAge BC*21 + 20. A further evaluation of baseline data was performed using Horvath’s mAge prediction formula based on 353 CpGs [9], with 334 CpGs available.

### Statistical analysis

This analysis's primary aim was to examine the association between age and mAge, calculated according to the prediction formula based on 240 CpGs (217 CpGs detected) [11]. Secondary aims included the association between mAge, fat deposits and metabolic blood biomarkers. Finally, we examined 18-month differences in mAge between intervention diet groups and other sub-groups, and differences in observed mAge change and assumed expected change. Continuous variables are presented as means ± standard deviations. Nominal variables are expressed as numbers and percentages. The Kolmogorov–Smirnov test was used to determine the variable's distribution. Pearson and Spearman's tests were used to examine the correlation between normally and not normally distributed variables, respectively. Differences between groups were tested using T-test, Mann–Whitney (for 2 groups comparisons)), Analysis of Variance (ANOVA) or Kruskal Wallis. Within group changes (baseline—T0 vs. end of the intervention—T18) were tested using Paired samples T-test.

For the association between baseline variables and methylation age acceleration, we used the standardized residual of mAge accounting for chronological age, reflecting the difference between methylation aging and chronological aging (“mAge deviation”, Additional file 6: Supplementary methods). Sex-specific tertiles of mAge deviation were evaluated using between group comparisons detailed above. To assess 18-m change in mAge, we examined 18-month absolute mAge change (mAge at T18—mAge at baseline = ∆mAge). For adjustments, we used multiple linear regression models. Although the mAge change was normally distributed, we presented the un-even subgroups (weight and liver status) as medians, 25th and 75th percentiles due to large standard deviations.

In order to examine differences between observed mAge at the end of the intervention, and the expected mAge resulting in case of no intervention occurred, we used the linear regression prediction formula generated from baseline correlation between age and mAge: *y* = 23.73 + 0.75 × baseline age plus the interval time between blood draws (24 month, a constant for all participants).

Statistical analysis was performed using SPSS (version 26.0) software. Statistical significance was set for *p* ≤ 0.05, two-sided. Plots were constructed using GraphPad Prism 7.

## Supplementary Information

**Additional file 1: Table S1**. Sensitivity analysis: baseline tertiles – men only.

**Additional file 2: Table S2**. MV models for assessing the association between mAge deviation and intrahepatic fat.

**Additional file 3: Table S3**. A summarize of the baseline correlations of mAge with adiposity and fat deposits according to two mAge prediction formulas.

**Additional file 4**. Supplementary results.

**Additional file 5: Figure S1**. Difference between the observed mAge at the end of the intervention and the assumed expected mAge difference.

**Additional file 6**. Supplemental methods.

## Data Availability

All data generated or analyzed during this study are included in this published article [and its supplementary information files].

## Abbreviations

BMI: Body mass index
DSAT: Deep subcutaneous adipose tissue
HbA1c: Hemoglobin A1c
HDL c: High-density lipoprotein cholesterol
HOMA IR: Homeostatic model assessment of insulin resistance
IHF: Intrahepatic fat
LF: Low fat
MED: Mediterranean
mAge: Methylation age
QC: Quality control
SSAT: Superficial subcutaneous adipose tissue
VAT: Visceral adipose tissue
WC: Waist circumference
