# Supplementary material for: Lifestyle weight-loss intervention may attenuate methylation aging: the CENTRAL MRI randomized controlled trial
Source: Clin Epigenetics. 2021 Mar 4;13:48. doi: 10.1186/s13148-021-01038-0 (PMC7934393; doi:10.1186/s13148-021-01038-0)
Supplement: Supplementary file 1 — Additional file 1: Table S1. Sensitivity analysis: baseline tertiles – men only. [file 13148_2021_1038_MOESM1_ESM.docx]

**Additional file 1: Table S1**

Sensitivity analysis: baseline tertiles – men only

|  | **Low**  **Tertile**  **(≤-0.44)**  **n=36** | **Intermediate tertile**  **(-.43 - .46)**  **n=38** | **High tertile**  **(≥.47)**  **n=36** | **p between tertiles^1^** | **p between extreme tertiles^2^** |
| --- | --- | --- | --- | --- | --- |
| Age, years | 47.1±10.7 | 50.5±8.8 | 47.2±8.7 | 0.20 | 0.98 |
| mAge, years | 56.1±7.8 | 62.0±6.6 | 62.2±6.9 | **<0.001** | **0.001** |
| Weight, kg | 90.0±9.2 | 91.0±11.4 | 93.8±10.1 | 0.26 | 0.1 |
| BMI, kg/m^2^ | 29.7±2.6 | 29.9±2.9 | 30.8±3.0 | 0.29 | 0.13 |
| WC, cm | 105.7±6.9 | 106.5±7.5 | 109.3±6.4 | ***0.05*** | **0.019** |
| VAT, cm2 | 170.2±51.3 | 171.0±56.6 | 200.9±68.1 | ***0.08*** | **0.047** |
| VAT proportion, % | 35.1±10.5 | 34.5±9.2 | 36.8±11.0 | 0.59 | 0.49 |
| DSAT, cm^2^ | 200.7 ±65.4 | 213.6±79.4 | 216.5±65.7 | 0.059 | 0.32 |
| DSAT proportion, % | 39.7±6.0 | 41.2±5.8 | 39.3±6.5 | 0.39 | 0.79 |
| SSAT, cm^2^ | 127.3±49.2 | 124.3±45.1 | 129.7±42.6 | 0.88 | 0.82 |
| SSAT proportion, % | 25.2±6.3 | 24.3±5.5 | 23.8±5.9 | 0.61 | 0.34 |
| IHF^3^, % | 7.7±7.7 | 9.9±10.0 | 14.3±11.2 | **0.02** | **0.004** |
| Fasting glucose, mg/dL | 101.2±10.4 | 111.3±19.1 | 108.8±17.5 | **0.02** | **0.03** |
| HOMA IR^4^ | 3.9±2.2 | 5.0±3.5 | 6.1±4.3 | **0.04** | **0.02** |
| HbA1c, % | 5.5±0.4 | 5.7±0.6 | 5.6±0.5 | 0.28 | 0.33 |

Data are mean±SD. ^1^ ANOVA or Kruskal Wallis ^2^ T-test or Mann-Whitney. BMI, body mass index; DSAT, deep subcutaneous adipose tissue; HbA1c, hemoglobin A1c; HOMA IR, Homeostatic Model Assessment of Insulin Resistance; IHF, intrahepatic fat; SSAT, superficial subcutaneous adipose tissue; VAT, visceral adipose tissue; WC, waist circumference.
