# Supplementary material for: Lifestyle weight-loss intervention may attenuate methylation aging: the CENTRAL MRI randomized controlled trial
Source: Clin Epigenetics. 2021 Mar 4;13:48. doi: 10.1186/s13148-021-01038-0 (PMC7934393; doi:10.1186/s13148-021-01038-0)
Supplement: Supplementary file 2 — Additional file 2: Table S2. MV models for assessing the association between mAge deviation and intrahepatic fat. [file 13148_2021_1038_MOESM2_ESM.docx]

**Additional file 2: Table S2**

MV models for assessing the association between mAge deviation and intrahepatic fat

|  | **β** | **p-value** |
| --- | --- | --- |
| Model 1+ hypertension^1^ | 0.235 | 0.011 |
| Model 1+ type 2 diabetes^2^ | 0.225 | 0.014 |
| Model 1+ Metabolic syndrome | 0.23 | 0.013 |
| Model 2+ hypertension | 0.238 | 0.013 |
| Model 2+ type 2 diabetes^2^ | 0.228 | 0.017 |
| Model 2+ Metabolic syndrome | 0.234 | 0.015 |
| Model 3+ hypertension^1^ | 0.237 | 0.014 |
| Model 2+ type 2 diabetes^2^ | 0.228 | 0.017 |
| Model 3+ Metabolic syndrome | 0.234 | 0.016 |

^1^ Systolic blood pressure >= 130 or diastolic blood pressure >= 85 or the report on any anti-hypertensive medication.

^2^ Calculated as fasting glucose >126 mg/dL or HbA1c >6.5% or the report on any oral glycemic control medication.

Model 1: Standardized residuals – adjusted for sex

Model 2: Standardized residuals – adjusted for sex and weight

Model 3: Standardized residuals – adjusted for sex, weight, and age
