# Supplementary material for: Lifestyle weight-loss intervention may attenuate methylation aging: the CENTRAL MRI randomized controlled trial
Source: Clin Epigenetics. 2021 Mar 4;13:48. doi: 10.1186/s13148-021-01038-0 (PMC7934393; doi:10.1186/s13148-021-01038-0)
Supplement: Supplementary file 3 — Additional file 3: Table S3. A summarize of the baseline correlations of mAge with adiposity and fat deposits according to two mAge prediction formulas. [file 13148_2021_1038_MOESM3_ESM.docx]

**Additional file 3: Table S3**

A summarize of the baseline correlations of mAge with adiposity and fat deposits according to two mAge prediction formulas

|  | **mAge (240 CpGs)** | | **mAge (353 CpGs)** | |
| --- | --- | --- | --- | --- |
|  | **r^*^** | **p** | **r^*^** | **p** |
| Weight, kg | -0.15 | 0.11 | -0.12 | 0.18 |
| Waist circumference, cm | 0.07 | 0.44 | 0.06 | 0.49 |
| Visceral adipose tissue, cm^2^ | **0.38** | **<0.001** | **0.37** | **<0.001** |
| Visceral adipose tissue proportion, % | **0.41** | **<0.001** | **0.42** | **<0.001** |
| Deep Subcutaneous adipose tissue, cm^2^ | -0.05 | 0.56 | -0.09 | 0.32 |
| Deep Subcutaneous adipose tissue proportion, % | **-0.27** | **0.003** | **-0.24** | **0.007** |
| Superficial Subcutaneous adipose tissue, cm^2^ | **-0.23** | **0.01** | **-0.23** | **0.01** |
| Superficial Subcutaneous adipose tissue proportion, % | **-0.38** | **<0.001** | **-0.45** | **<0.001** |
| Liver fat, % | 0.11 | 0.23 | 0.11 | 0.25 |

*Pearson test for 2 normally distributed variables, Spearman test if at least one is not.
