# Supplementary material for: Lifestyle weight-loss intervention may attenuate methylation aging: the CENTRAL MRI randomized controlled trial
Source: Clin Epigenetics. 2021 Mar 4;13:48. doi: 10.1186/s13148-021-01038-0 (PMC7934393; doi:10.1186/s13148-021-01038-0)
Supplement: Supplementary file 4 — Additional file 4. Supplementary results. [file 13148_2021_1038_MOESM4_ESM.docx]

**Additional file 4: supplementary results**

Supplementary results: Subgroup analysis by chronological age

In a subgroup analysis by chronological age, participants above median age (47.8 years) in the low fat (LF) group significantly increased their methylation age (mAge) by 1.0±2.1 years (p=0.02 vs. baseline), while older participants from the Mediterranean/low carbohydrate (MED/LC) group had a non-significant change in mAge after 18 months (0.5±1.9 years, p=0.13 vs. baseline), with no significant difference between the groups (p=0.35). In participants below the median age, both diet groups increased significantly their mAge (LF: 1.6±1.7 years, p<0.001; MED/LC: 1.2±1.8 years, p=0.002 vs. baseline), with no significant difference between groups (p=0.33).

Supplementary results: Observed mAge change (∆mAge) in relation to the predicted mAge change

As predicted by linear regression generated at baseline, the assumed expected mAge after intervention for the entire cohort was 61.6±7.0 years, thus, expected aging of 1.3±2.8 years (~15.7 months). As reported, participants actual mAge increased by 1.1±1.9 (~12.9 months) years from baseline (observed mAge). In the subgroup with healthy liver status at the end of the intervention, the observed mAge was significantly lower by 10.3 months than the assumed expected (p=0.001), as mAge increased by 8.3±23.0 months from baseline to the end of intervention, while the expected mAge for the same set of participants would have been increased 18.6±31.9 months.

In a sub analysis among men above the median age, the observed mAge was significantly lower than the assumed expected (p=0.048), where mAge increased by 7.1±23.4 months from baseline, while the expected mAge change was assumed as 14.8±35.8 months. In weight successors, among men above median age, a marginal difference was observed between the observed and expected mAge change (4.6±25.6 months vs. 16.9±31.8 months, respectively; p=0.08(. For men above median age with normal liver fat%, lower mAge change was observed (2.7±23.7 months), as compared to the expected mAge change (20.5±32.7 months; p=3.0*10^-4^).
