# Supplementary material for: Lifestyle weight-loss intervention may attenuate methylation aging: the CENTRAL MRI randomized controlled trial
Source: Clin Epigenetics. 2021 Mar 4;13:48. doi: 10.1186/s13148-021-01038-0 (PMC7934393; doi:10.1186/s13148-021-01038-0)
Supplement: Supplementary file 5 — Additional file 5: Figure S1. Difference between the observed mAge at the end of the intervention and the assumed expected mAge difference. [file 13148_2021_1038_MOESM5_ESM.docx]

**Additional file 5: Figure S1**

Difference between the observed mAge at the end of the intervention and the assumed expected mAge difference


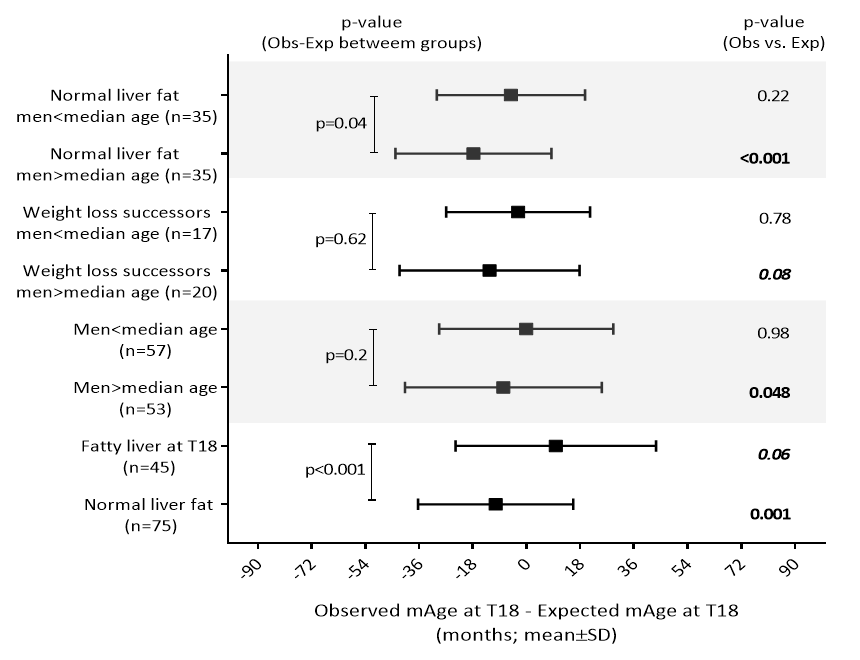


Data presented as mean±SDs for the difference between mAge at T18 and the expected mAge at T18. Between groups differences were tested using independent samples T test or Mann-Whitney test. Paired test was used for observed (Obs) vs. expected (Exp) mAge, both by T18.
