# Supplementary material for: Lifestyle weight-loss intervention may attenuate methylation aging: the CENTRAL MRI randomized controlled trial
Source: Clin Epigenetics. 2021 Mar 4;13:48. doi: 10.1186/s13148-021-01038-0 (PMC7934393; doi:10.1186/s13148-021-01038-0)
Supplement: Supplementary file 6 — Additional file 6. Supplemental methods. [file 13148_2021_1038_MOESM6_ESM.docx]

**Additional file 6: supplemental methods**

Supplementary methods: Technical description of the scanning procedure, abdominal fat depots and IHF% acquisition and Clinical measurements

*MRI assessment of fat deposits:*

The scanner utilized a 3D modified DIXON (mDIXON) imaging technique without gaps (2mm thickness and 2mm of spacing), fast-low-angle shot (FLASH) sequence with a multi-echo two-excitation pulse sequence for phase-sensitive encoding of fat and water signals (TR,3.6ms; TE1,1.19ms; TE2,2.3ms; FOV 520×440×80mm; 2×1.4×1mm voxel size). Four images of the phantoms were generated, including in-phase, out-phase, fat and water phase. A breath-hold technique was used to avoid motion artifacts when chest and abdomen were scanned. All MRI tests were performed after fasting 2+ hours to avoid artifacts of fat-disruption in the intestines. In all simultaneous fat depots quantification and comparisons, observers were blinded to point of measurement and group treatment.

Abdominal fat depots were quantified using a MATLAB-based semi-automatic program with a continuous line drawn over the fascia superficialis to differentiate deep-subcutaneous adipose tissue (SAT) and superficial-SAT. Mean visceral adipose tissue (VAT), deep-SAT and superficial-SAT areas were calculated from three axial slices: L5-S1, L4-L5 and L2-L3. IHF content was calculated using the PRIDE software from Philips Medical Systems. We calculated mean percentages from 2D slices (3cm intervals divided into quarters) by utilizing the region of interest (ROI) approach [1], examining tissue densities (fat/fat+water) based on the fat ratio calculation. We determined mean fat percentage for each slice and quarter, and for the entire liver.

*Clinical measurements:*

Height was measured at baseline, to the nearest millimeter by using a standard wall-mounted stadiometer. WC was measured at baseline and 18 months thereafter to the nearest millimeter with an anthropometric measuring tape; the measurement was made half-way between the last rib and the iliac crest. Body weight was measured monthly without shoes to the nearest 0.1kg. Fasting blood samples were taken at baseline and 18 months thereafter at 8:00am, and were stored at ‑80°C.

Fasting plasma glucose (FPG) was measured by Roche GLUC 3 (hexokinase method). Plasma insulin was measured with an enzyme immunometric assay [Immulite automated analyzer, Diagnostic Products, coefficient of variation (CV) =2 .5%]. Serum total cholesterol (CV = 1.3%), high-density-lipoprotein cholesterol (HDL-c), low-density-lipoprotein (LDL) cholesterol, and triglycerides (CV = 2.1%) were determined enzymatically with a Cobas 6000 automatic analyzer (Roche). Plasma leptin levels were assessed by ELISA (Mediagnost, CV = 2.4%). All biochemical analyses were performed at the laboratories of University of Leipzig Medical Center, Germany.

Supplementary methods: mAge deviation

Standardized residuals of chronological age and mAge were saved from linear regression function of mAge ~ chronological age. Then, sex specific tertiles were constructed, considering the specific tertiles for men and women separately (men Lowest tertile: men: ≤-0.44; women ≤ -0.77; intermediate tertile: men: -0.43 to 0.46, women: -0.76 to 0.55; Highest tertile: men: 0.47+, women: 0.56+). Lowest tertile of standardized residuals represent the values that are below the prediction line, thus generating a negative deviation from the prediction line. Highest tertile of the standardized residual represent values that have positive deviation from the prediction live (above the line). In most cases, values below prediction line represent higher chronological age than mAge, and values above prediction line represent lower chronological age than mAge.

Supplementary Figure 2: mAge positive and negative deviation


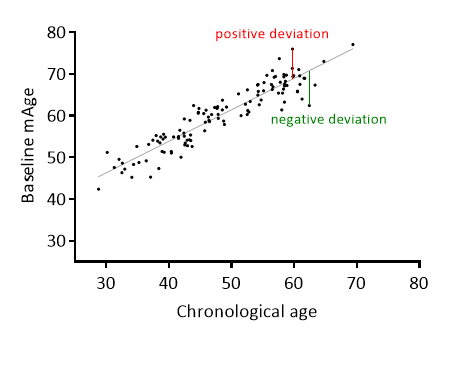


**References**

1. Schuchmann S, Weigel C, Albrecht L, Kirsch M, Lemke A, Lorenz G, et al. Non-invasive quantification of hepatic fat fraction by fast 1.0, 1.5 and 3.0 T MR imaging. Eur J Radiol. 2007;62(3):416-22.
